# A reinterpretation of the gap fraction of tree crowns from the perspectives of computer graphics and porous media theory

**DOI:** 10.3389/fpls.2023.1109443

**Published:** 2023-02-06

**Authors:** Yunfeng Zhu, Dongni Li, Jiangchuan Fan, Huaiqing Zhang, Markus P. Eichhorn, Xiangjun Wang, Ting Yun

**Affiliations:** ^1^ School of Information Science and Technology, Nanjing Forestry University, Nanjing, China; ^2^ National Engineering Research Center for Information Technology in Agriculture, Beijing, China; ^3^ Research Institute of Forestry Resource Information Techniques, Chinese Academy of Forestry, Beijing, China; ^4^ School of Biological, Earth and Environmental Sciences, University College Cork, Cork, Ireland; ^5^ Environmental Research Institute, University College Cork, Cork, Ireland; ^6^ Rubber Research Institute, Chinese Academy of Tropical Agricultural Sciences, Haikou, China; ^7^ Forestry College, Nanjing Forestry University, Nanjing, China

**Keywords:** volume-based gap fraction, porous media theory, fine geometric characterization, equivalent leaf thickness, computer graphics

## Abstract

The gap fraction (GF) of vegetative canopies is an important property related to the contained bulk of reproductive elements and woody facets within the tree crown volume. This work was developed from the perspectives of porous media theory and computer graphics techniques, considering the vegetative elements in the canopy as a solid matrix and treating the gaps between them as pores to guide volume-based GF_vol_ calculations. Woody components and individual leaves were extracted from terrestrial laser scanning data. The concept of equivalent leaf thickness describing the degrees of leaf curling and drooping was proposed to construct hexagonal prisms properly enclosing the scanned points of each leaf, and cylinder models were adopted to fit each branch segment, enabling the calculation of the equivalent leaf and branch volumes within the crown. Finally, the volume-based GF_vol_ of the tree crown following the definition of the void fraction in porous media theory was calculated as one minus the ratio of the total plant leaf and branch volume to the canopy volume. This approach was tested on five tree species and a forest plot with variable canopy architecture, yielding an estimated maximum volume-based GF_vol_ of 0.985 for a small crepe myrtle and a minimal volume-based GF_vol_ of 0.953 for a sakura tree. The 3D morphology of each compositional element in the tree canopy was geometrically defined and the canopy was considered a porous structure to conduct GF_vol_ calculations based on multidisciplinary theory.

## Introduction

The gap fraction (GF) of a tree crown, otherwise known as crown porosity, is of high importance for characterizing forest habitats. GF is not only a key parameter for accurately quantifying the leaf area index (LAI) but also an important factor for better understanding plant–environment exchange processes such as photosynthetic light utilization (Carraretto et al., 2013), plant transpiration and canopy precipitation interception (Baldocchi and Wilson, 2001). Lack of airflow in a plant canopy can create a humid microclimate, while an increase in GF facilitates airflow within the canopy, increases exposure of the interior foliage elements to solar irradiance, reduces internal shadow areas, increases the number and penetration of sunflecks, and reduces prevalence of fungal infections. In addition, GF can be a biological indicator for determining the number of fallen leaves and the leaf area density (Solberg, 2010). Therefore, accurate retrieval of the GF of canopies is of central importance to a wide variety of physiological, climatological, and biogeochemical studies.

Existing methods have been developed based on modern optical imaging and laser-scanning techniques. The methods for calculating GF can be divided into two categories. The first category is image processing-based methods (Ryu et al., 2012); these methods involve upwards-capturing hemispherical photographs (HPs) (Lang et al., 2010), derived hemisphere images from scanned points (Grotti et al., 2020) or tree models (Woodgate et al., 2015) of the studied forest established through stereographic projection. Notably, GF_img_ can be calculated as the number of sky pixels (excluding pixels of crown cover) to the total number of image pixels (Hwang et al., 2016). The other category includes optical transmission model-based methods (Ponce de León and Bailey, 2019). In these methods, the number of emitted laser beams within the angular range covering the target tree is counted, and the travel distance of beams in the tree crown is determined. Moreover, laser hits are divided into two classes, namely, complete interception and complete penetration without blockage by vegetative elements, and this information is coupled with the designed formulas to retrieve the gap fraction of the target tree crown (Li et al., 2017; Wen et al., 2019).

Despite the numerous approaches proposed to detect gap fractions within tree crowns, tree crowns have geometrical peculiarities and variable morphologies, leading to variations in the results of the aforementioned methods with changes in the viewing angles and observational positions (Béland et al., 2014). Usually, the image processing-based methods used to analyse HPs yield different GF_img_ magnitudes for different ring segments corresponding to different zenith angles in a forest plot, and uniform criteria or mechanisms to explain the differences in the results from various captured HPs in a forest plot do not exist (Lang et al., 2010). Likewise, optical transmission model-based methods are sensitive to light emission angles. Partial interception and gap identification from light detection and ranging (LiDAR) data at a fixed emission angle are related to the horizontal and vertical distributions of the crown mass considering the stem base position at a given angle (Milenković et al., 2017); thus, a statistically significant sample of trees is needed, and it is difficult to quantify full tree crowns with asymmetrical phenotypic characteristics and allometric growth properties. In addition, some statistical methods (Zou et al., 2020) are applicable only to large-scale forestlands, and they require large amounts of data analysis (Kong et al., 2022) or standardization of the distance and orientation of sample data to compensate for the deviations of individual specimens; therefore, these methods are not suitable for single trees without guidance from prior analyses of comparatively larger forest plots with large populations of the same species.

A porous medium is a material containing pores (voids). The skeletal portion of the material is often called the “matrix” or “frame”. The pores are typically filled with a fluid (liquid or gas), and the skeletal material is usually a solid (Volinia et al., 2006). Many natural substances, such as rocks, soil, and biological tissues, e.g., bones, wood, cork, and cotton, can be considered porous media because their intrinsic properties are similar to those of porous media. As an important biological element on Earth, tree crowns comprise stems, foliage and reproductive structures that remain nearly static relative to the ground over time, with air currents and sunlight travelling through the canopy. Therefore, the tree crown can be regarded as a multiphasic and porous medium when considering both photosynthetic and non-photosynthetic parts of the canopy as skeletal materials and treating the gaps between the plant materials in the tree crown as pores allowing airflow circulation and light penetration.

Following the indicator of the void fraction (Jones and Zuber, 1975) in porous media theory (Ehlers, 2002), which is defined as the fraction of the channel volume that is occupied by the gas phase to the total volume, the concept of void fractions can be extrapolated to define the volume-based GF_vol_ of tree crowns by assessing the volumetrically occupied branch and leaf aggregations of the tree crown; additionally, the residual volume proportion of void space (gaps) between the vegetative elements within the tree crown can be defined. This volume-based GF_vol_ is a normalized variable that reflects the overall spatial volume of constituents in the tree crown. Moreover, the synergetic use of computer graphics techniques (Oishi et al., 2012) to mine the local geometrical structural features of the vegetative tissues from the scanned point clouds (Van Leeuwen et al., 2013) affords us an opportunity to portray in detail the 3D morphologies of plant organs. Hence, the volume-based GF_vol_ can be used to characterize the inner structure of the tree crown based on the total volume estimates of plant materials and pore space in the tree crown. This method differs from approaches to estimating GF based on the appearance of the tree crown in captured images or point cloud projections, which are variable and unstable depending on the dimensionality and observation point limitations.

Based on the concept of volume-based gap fraction, a methodology for calculating the volume-based GF_vol_ is established in this study based on LiDAR data derived from porous media theory, and computer graphics techniques are applied to obtain visual traits at a fine scale. Individual leaves are extracted from point clouds based on the proposed segmentation algorithm, and then moving least squares and cubic polynomial fitting methods are employed to eliminate noise and to extract complete edges of each leaf. The new definition of the equivalent leaf thickness depicting the curl and droop degrees of leaf lamina and the utilization of hexagonal prisms and cylinders that envelop the fitted leaf and branch points, respectively, support the calculation of the ratio of the total volume of vegetative elements in the tree crown to the crown volume measured. All the factors contributing to a volume-based GF_vol_ calculation are applied to the tree crowns of five different species and a forest plot, and the results are compared with those of existing approaches.

## Materials and methods

### Study site and data collection

The study sites were on the campus of Nanjing Forestry University (32 08’N, 118 81’E, WGS-84) in Nanjing City, Jiangsu Province, and a rubber tree plantation (19 32’N, 109 2’E, WGS-84) in Danzhou city on Hainan Island, China. Trees from four isolated stands, namely, a sakura tree (*Prunus* sp.*))*, a crepe myrtle tree (*Lagerstroemia indica* (L.) Pers), a magnolia tree (*Magnolia figo* (Lour) DC.) and a ginkgo (*Ginkgo biloba*), located on our campus, together with a rubber tree (*Hevea brasiliensis* Müll. Arg.) of clone CATAS 7-20-59 and a pure rubber tree plot of clone PR 107 located in Danzhou city, were selected as the experimental targets.

Complete point clouds of the five trees and a forest plot were obtained using a Leica C10 terrestrial laser scanning (TLS) system in October 2019. The Leica C10 instrument was a 532-nm phase-based scanner with a 360°×270° upwards field of view and a laser scanning rate of 5000 points per second. The Leica C10 scanner yields a minimum distance between consecutive beams of approximately 0.4 mm and a target measurement accuracy of ±1.5 mm at 3 m from the instrument, with a scanning speed of up to 50,000 points/second. According to the different heights of the five experimental trees, distances of 3 m from the three smaller trees (sakura, crepe myrtle and magnolia trees) and 5 m from the two taller trees (ginkgo and rubber trees) to the laser scanner were set, and two side lateral scans from opposite sides of each tree crown pointing towards the centre of the tree crown and a multiscan set up in the forest plot were used to achieve complete phenotypic characteristic scanning of the target trees and the forest plot.

Many growth properties of the studied trees were manually measured *in situ* and are shown in **Table 1**. The tree height was measured using a Vertex IV hypsometer, and the diameter at breast height (DBH) was measured over the outside bark using a diameter tape at 1.37 m above the ground floor. The leaf number was counted by three students alongside the tree crowns according to the relations among foliage clumps growing on different branches, and the crown width was measured by tapes in two horizontally perpendicular directions from the location of each tree top.

**Table 1:** Illustration of the growth attributes and the number of scanned points for the five studied trees.

	Small trees		Tall trees		
Experimental trees	Crepe myrtle	Sakura	Magnolia	Rubber	Ginkgo
Height (m)	2.52	3.97	3.42	16.15	7.34
DBH (m)	0.07	0.10	0.09	0.21	0.11
Crown width (N‒S/W‒E) (m)	2.69/1.98	2.82/3.17	1.72/1.79	6.86/6.24	4.43/3.31
Number of scanned points (wood/leaf)	35275/ 58251	80526/ 321729	46783/ 116150	583560/ 120193	413260/ 50549
Equivalent thickness of the leaf (side view/parallel view along the midrib) (cm)	0.63 ± 0.39/ 1.34 ± 0.93	0.85 ± 0.51/ 1.55 ± 0.73	1.14 ± 0.77/ 2.55 ± 1.30	0.86 ± 0.56/ 1.93 ± 1.11	0.92 ± 0.70/ 2.14 ± 1.22

### Data pre-processing

**Figure 1:**
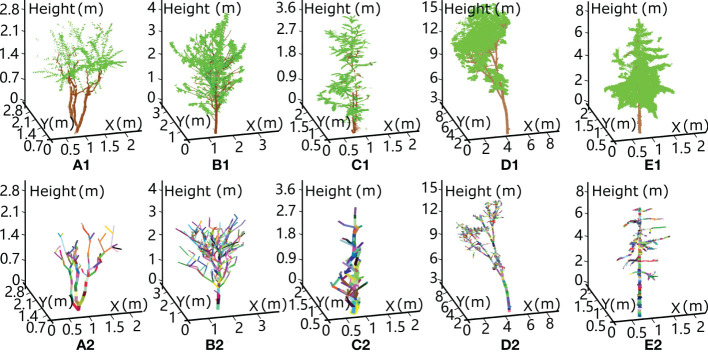
Schematic diagram showing wood–leaf separation and branch skeleton reconstruction from terrestrial laser scanning data for five experimental trees: A **(A)** crepe myrtle tree, **(B)** sakura tree, **(C)** magnolia tree, **(D)** rubber tree and **(E)** ginkgo tree. The first row lists the classified leaf and branch points represented in green and brown, respectively. The second row shows the reconstruction results of the branch segments based on the cylinder model fitting algorithm.

The raw point cloud data were processed using a wood–leaf segmentation algorithm (Yun et al., 2016) to perform leaf and wood scanned point classification. The wood-leaf segmentation algorithm manually defines the salient features of the point clouds of branches (approaching linear features with univariate morphological scaling yielding spatial stretching variations) and leaves (approaching plane features with bivariate morphological scaling relationships describing the size variation of a thin flat sheet) based upon the eigenvalues of the covariance matrix calculated at each point and its neighbouring points. Consequently, machine learning algorithms, e.g., Gaussian classifiers or support vector machines (SVMs), are employed to discriminate the category of each point based on the derived features. Furthermore, modelling of branches with a sequence of fitted cylinders with an adaptive radius scheme (Huang et al., 2020) was conducted for wood volume estimates. The results for wood–leaf separation and branch segment reconstruction are shown in **Figure 1** for five experimental trees.

### Individual leaf segmentation

An individual leaf segmentation algorithm was designed here for the extracted leaf points after wood-leaf point segmentation. The leaf morphologies are similar to ellipses or fans, with local patches manifesting planar geometric invariants. Hence, a scanned point on the leaf surface associated with its neighbouring points is characterized by planarity and continuousness as dominant features, reflecting that an isotropic distribution of neighbouring points is located along a great circle, with the spherical neighbourhood centred at a true centre point. Based on the above principle, the specific steps for extracting the centre point of each leaf surface were established as follows.

First, the spherical neighbourhood of each leaf point *p*_*i*_(*x*_*i*_,*y*_*i*_,*z*_*i*_)∈*P* with the assigned radius equal to the three quarters of the average half leaf width was determined. Second, it was determined whether a great circle, i.e., a circle that intersects the spherical neighbourhood and passes through the centre point *p_i_* of the sphere, exists, with a small distance between the great circle *Ax*_*i*_+*By*_*i*_+*Cz*_*i*_=*D* and the *N*_1_ neighbour points *p*_*j*_(*x*_*j*_,*y*_*j*_,*z*_*j*_)∈*P* of *p_i_*. The specific equation is as follows:

where *Ax_i_* + *By_i_* + *Cz_i_* = *D* is the great circle plane determined by employing a least square fitting algorithm (Bates and Watts, 1988) for neighbouring point set *p_j_* and passing through the centre point *p_i_A, B* and *C* are the components of the normal vector of the great circle plane.

Third, the true centre point of the leaf surface is associated with a unanimously directional normal vector with surrounding points in the spherical neighbourhood on the same leaf surface. The following equation was adopted to calculate the normal vector of the centre point


$$\underset{{\vec{n}}_{i}}{\arg\min}\sum_{j=1}^{N_{1}}{\left(w_{j}^{T}{\vec{n}}_{i}\right)}^{2}e^{-{\left(w_{j}^{T}{\vec{n}}_{i}\right)}^{2}}$$

where *w*_*j*_=*p*_*j*_−*p*_*i*_ The true ${\vec{n}}_{i}$ of the centre point *p*_*i*_ that minimizes the result of equation (2) can be calculated. Then, the true centre point of each leaf surface should correspond to a normal vector of consistent direction with those of neighbouring points, i.e.,


$$\sum_{j=1}^{N_{1}}\frac{\arccos\left({\vec{n}}_{i}\cdot {\vec{n}}_{j}\right)}{N_{1}}<threshold_{2}$$

$${\vec{n}}_{j}$$

is the normal vector of the neighbouring point *p_j_*

If the points in the spherical neighbourhood satisfy the two conditions mentioned above, all the neighbour points are projected onto the great circle defined in equation (3). Then, the following equation is used for a projection transformation from the original neighbouring points *p*_*j*_(*x*_*j*_,*y*_*j*_,*z*_*j*_) to the projected points


$${p^{\prime }}_{j}\left({x^{\prime }}_{j},{y^{\prime }}_{j},{z^{\prime }}_{j}\right)$$

on the great circle.


(3) $$\{\begin{array}{c} {x^{\prime }}_{j}=\frac{\left(B^{2}+C^{2})x_{j}-A(By_{j}+Cz_{j}+D\right)}{A^{2}+B^{2}+C^{2}}\\ {y^{\prime }}_{j}=\frac{\left(A^{2}+C^{2})y_{j}-B(Ax_{j}+Cz_{j}+D\right)}{A^{2}+B^{2}+C^{2}}\\ {z^{\prime }}_{j}=\frac{\left(A^{2}+B^{2})z_{j}-C(Ax_{j}+By_{j}+D\right)}{A^{2}+B^{2}+C^{2}} \end{array}$$

Consequently, the great circle is divided into 40 space segments with equal areas through four concentric rings and eight lines emitted from the circle centre with the same angular separation. The projected points uniformly distributed in the great circle can be evaluated based on the normalized standard deviation derived from the following equation:


$$\sigma^{\prime }=\sqrt{\frac{\sum_{d=1}^{40}{\left(num_{d}^{p^{\prime }}-\mu \right)}^{2}}{40}}/\mu$$

where *μ* is the average number of projected points in 40 segments of the great circle and $num_{d}^{p^{\prime }}$ represents the number of projected points *p*′_*j*_(*x*′_*j*_,*y*′_*j*_,*z*′_*j*_) in the *d* t*h* segment. If *σ* ^′^≤*threshold*_3_, we consider the neighbouring points well-proportioned in each segment around the centre point and assume that they homogeneously cover the whole great circle, as *p_j_* is located in the centre of an integral leaf surface. After determination of the centre points of every leaf surface, the 3D watershed method (Lin et al., 2003) and the DBSCAN algorithm (Latella et al., 2021) were employed for individual leaf extraction from the scanned points of the photosynthetic part of the canopy.

### Physical and equivalent leaf thicknesses

The physical thickness of a leaf blade is usually a few millimetres, which is related to the number of anatomical features (e.g., cell walls and chloroplasts), biomass partitioning, net productivity and crop response to water deficits (Pauli et al., 2017). In addition, the zenith angle and azimuth angle of the blades vary with different growth stages, environmental conditions and physiological characteristics (Meng et al., 2013). For a single leaf, the efficiency of photosynthetic energy conversion or transpiration is related to the angle between the incident solar beams or air current and the normal vector of the leaf surface (Kitaya et al., 2000; Falster and Westoby, 2003). In principle, when the incoming light is perpendicular to the leaf surface with the largest illuminated leaf surface area or the greatest number of stomata engaged in gas exchange, the photosynthetic or transpiration efficiency is the highest, and the opposite holds for incoming solar light or airflow parallel to the leaf surface. Hence, a question arises: when the angle of incoming solar light or airflow is parallel to the leaf surface, such that only a few millimetres of leaf surface represented by the thickness of the leaf is exposed to light or airflow, does the leaf provide a sufficient photosynthetic area or stomata for light absorption and water vapour release?

**Figure 2:**
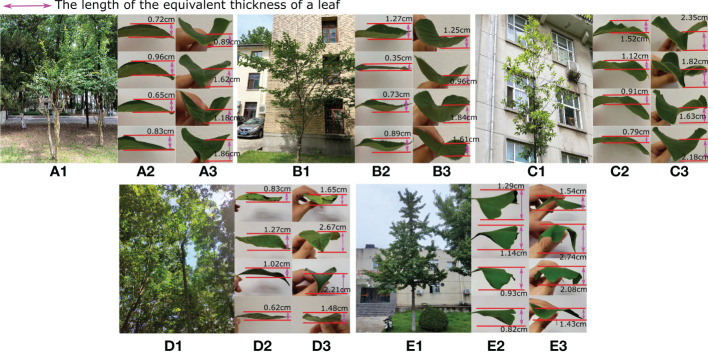
Sample leaves were collected from some test trees, namely, **(A)** crepe myrtle, **(B)** sakura, **(C)** magnolia, **(D)** rubber and **(E)** ginkgo tree, shown from the lateral side (a2-e2) and parallel to the leaf midrib (a3-e3). Each leaf displays different degrees of curling and drooping, which influence the equivalent leaf thickness, as indicated by the double-sided pink arrow.

In nature, leaf surfaces are distorted to varying degrees. The surface of a leaf is usually twisted, curly, droopy and even spiralled (Niklas, 1999). Many reasons can be given for these phenomena, such as the loss of water due to evaporation, the sustained absence of rain, sunburn in summer or hot weather, pests eating leaves, physiological diseases, the over-spraying of pesticides on leaves and the presence of rugged leaf veins. Here, we inferred that these distortions may be due to natural selection, causing the leaf surface to absorb more ambient light and to optimize respiration and transpiration with air currents from various directions. Therefore, when a leaf is observed from the side with a sightline parallel to the plane of the leaf surface, i.e., the lateral side **Figure 2** (a2-e2) or parallel to the leaf midrib **Figure 2** (a3-e3), the observed thickness is greater than the physical thickness of the leaf, as shown in **Figure 2**. Here, the observed thickness of a leaf that hinders airflow or sunlight from lateral side is defined as the equivalent leaf thickness. **Figure 2** shows different leaves of five tree species, namely, (a) crepe myrtle, (b) sakura, (c) magnolia, (d) rubber and (e) ginkgo tree, and an equivalent leaf thickness caused by curling and drooping of the leaf blades can be seen. We randomly sampled 100 leaves evenly distributed in each tree crown, and the ranges of the manually measured equivalent leaf thickness values for the target trees are shown in the last row of **Table 1** from lateral and parallel to the midrib perspectives.

### Calculation of the equivalent thickness and volume of each leaf

TLS can be used to acquire dense point clouds that describe in detail the phenotypic traits of spatial objects. Through two opposite scan registrations, integrated point clouds of each leaf in the canopy were obtained; here, a method based on computer graphics was used to estimate the equivalent thickness and volume of each leaf.

In the scanning process, trees were moved by wind, causing leaf and branch vibrations, which yield ghost points with position deviations for the scanned leaves and inevitably led to error propagation in the final leaf surface characterization. Therefore, it is necessary to constrain each leaf blade to an optimal fitting surface to eliminate the ghost points. Here, MLS (Lancaster and Salkauskas, 1981) was employed to process each leaf point after individual leaf point extraction.

**Figure 3:**
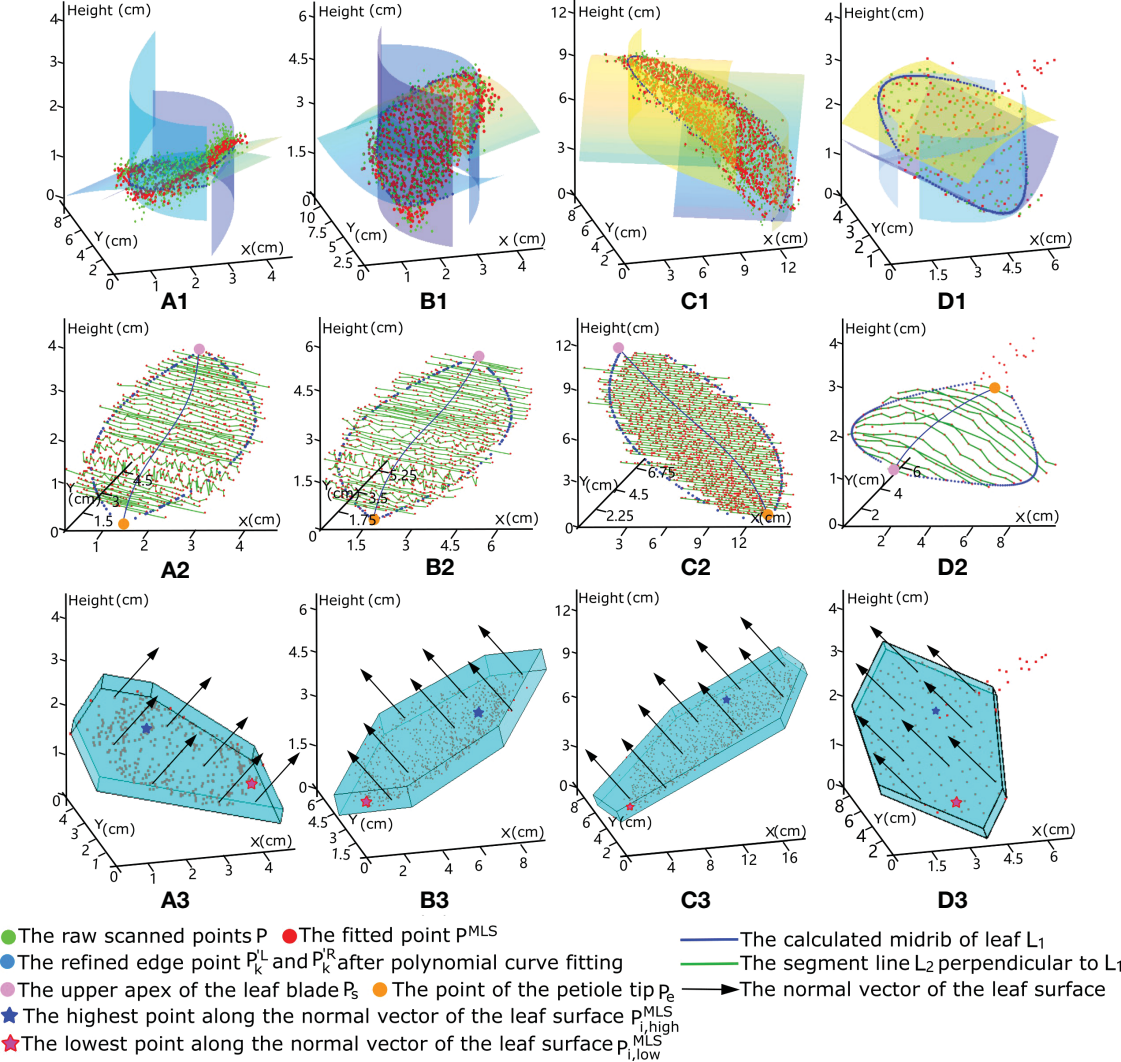
3D mock-up modelling process for certain leaves of different tree species using hexagonal prism envelopes to calculate the corresponding equivalent volume of each leaf blade. Each column shows the procedure of modelling a leaf of different tree species: **(A)** crepe myrtle leaf, **(B)** sakura tree, **(C)** magnolia leaf and **(D)** ginkgo leaf. The first row shows that the raw scanned points, represented in green, are fitted using moving least squares for noise removal and surface smoothing, with the fitted results represented by the red points. The second row shows that the leaf surface was divided into many segments according to green line *L_2_* perpendicular to leaf midrib *L_1_*, as shown by the blue line. The edge points of each segment on both sides refined using polynomial curve fitting are shown in blue. The third row shows that the constructed hexagonal prism envelope is modelled according to the refined edge points, with its height equal to the distance between the highest and the lowest fitted leaf points along the normal vector of the leaf surface.

After removal of noise to obtain the fitted points P of each smooth leaf surface, depiction of the real left and right edges of each leaf surface is necessary for leaf model construction. The two endpoints


$$p_{e}\left(x_{e},y_{e},z_{e}\right),\;p_{s}\left(x_{s},y_{s},z_{s}\right)\in P^{MLS}$$

in the direction of the midrib of the leaf surface that form the central axis *L*_1_ with the longest distance through the leaf surface can be easily identified, wherein *p*_*e*_ is the point of the petiole tip and *p*_*s*_ is the upper apex of the leaf blade (i.e., the top of the midrib), as shown by the orange and pink points in **Figure 3**, respectively. Then, the edge points of each leaf blade can be identified with a series of lines with uniform spacing perpendicular to the central axis *L*_1_ and intercepting the leaf scanned points, thus producing the corresponding left and right edge points, which are denoted as $p_{k}^{L}\left(x_{k}^{L},y_{k}^{L},z_{k}^{L}\right),\;p_{k}^{R}\left(x_{k}^{R},y_{k}^{R},z_{k}^{R}\right)\in P^{MLS}$ and *k*=1,2,3...*n*-1, respectively, as shown in **Figure 3**(a2), (b2), (c2) and (d2).

Similarly, as the environmental influences and intra-canopy occlusion affect the scanned data quality, the connection of the edge points (scanned points) at each side of the leaf usually forms an irregular curve, creating a rough edge profile. Hence, a cubic polynomial curve fitting method (Spitzer et al., 2006) was used to fit a nonlinear relationship between the independent *y_k_* variable and the dependent variables *x_k_* and *z_k_* with a *3*-degree polynomial for all the edge points at each side. The specific formula for the left- or right-side edge points is given as follows:


(5) $$F^{y\to x}\left(v_{b}^{y\to x}\right)=\frac{1}{2}{\sum_{k=1}^{n-1}\left[\sum_{b=0}^{3}v_{b}^{y\to x}{\left(y_{k}^{L}\right)}^{b}-x_{k}^{L}\right]}^{2};\;F^{y\to z}\left(v_{b}^{y\to z}\right)=\frac{1}{2}{\sum_{k=1}^{n-1}\left[\sum_{b=0}^{3}v_{b}^{y\to z}{\left(y_{k}^{L}\right)}^{b}-z_{k}^{L}\right]}^{2}$$

The fitted variables *x*′_*k*_ and *z*′_*k*_ of the left edge points ${P^{\prime }}_{k}^{L}\left({x^{\prime }}_{k}^{L},y_{k}^{L},{z^{\prime }}_{k}^{L}\right),k=1,2,3n-1$ can be presented as follows: ${x^{\prime }}_{k}^{L}=\sum_{b=0}^{3}v_{b}^{y\to x}{\left(y_{k}^{L}\right)}^{b}=v_{0}^{y\to x}{\left(y_{k}^{L}\right)}^{0}+v_{1}^{y\to x}{\left(y_{k}^{L}\right)}^{1}+v_{2}^{y\to x}{\left(y_{k}^{L}\right)}^{2}+v_{3}^{y\to x}{\left(y_{k}^{L}\right)}^{3}$ , ${z^{\prime }}_{k}^{L}=\sum_{b=0}^{3}v_{b}^{y\to z}{\left(y_{k}^{L}\right)}^{b}=v_{0}^{y\to z}{\left(y_{k}^{L}\right)}^{0}+v_{1}^{y\to z}{\left(y_{k}^{L}\right)}^{1}+v_{2}^{y\to z}{\left(y_{k}^{L}\right)}^{2}+v_{3}^{y\to z}{\left(y_{k}^{L}\right)}^{3}$ The partial derivatives of Formula (5), i.e., $\frac{\partial F^{y\to x}}{\partial v_{b}^{y\to x}}=0$ and $\frac{\partial F^{y\to z}}{\partial v_{b}^{y\to z}}=0$ , with respect to the calculated coefficients *v*_*b*_, *b*=0,1,2,3 can be used to minimize the error derived from curve fitting, and the optimal coefficients $v_{b}^{y\to x}$ and $v_{b}^{y\to z}$ can be obtained to determine the refined left-edge points ${P^{\prime }}_{k}^{L}\left({x^{\prime }}_{k}^{L},y_{k}^{L},{z^{\prime }}_{k}^{L}\right),k=1,2,3n-1$ with the fitted values ${x^{\prime }}_{k}^{L}$ and ${z^{\prime }}_{k}^{L}$ . A similar fitting operation is adopted for the edge points on the right side of each leaf. The refined edge points are highlighted in blue in **Figure 3**(a2), (b2), (c2) and (d2), which provide a complete representation of the leaf edge morphology.

### Enclosing each leaf using a hexagonal prism with equivalent leaf thickness

Because the most common leaf shapes are oval and elliptical, a hexagonal shape defined based on six spatial vertexes provides a favourable graphical presentation for approximating each leaf surface. Hence, for each leaf with *n*-1 refined left- and right-edge points $p_{k}^{L}\left(x_{k}^{L},y_{k}^{L},z_{k}^{L}\right),\;p_{k}^{R}\left(x_{k}^{R},y_{k}^{R},z_{k}^{R}\right)\in P^{MLS}$ and *k*=1,2,3...*n*-1, we followed the top-down order and chose two symmetrical edge points on each side with counting numbers of *round*((*n*−1)/4) and *round*(3(*n*−1)/4), which are represented as $p_{round\left(n/4\right)}^{L}$ , $p_{round\left(3n/4\right)}^{L}$ , $p_{round\left(n/4\right)}^{R}$ and $p_{round\left(3n/4\right)}^{R}$ and in conjunction with the two endpoints *p_e_* and *p_s_* of the midrib form the six vertices of the hexagonal base, as shown in **Figure 3**(a3), (b3), (c3) and (d3), where *round* () denotes the number in parentheses to the nearest integer.

Next, we calculated the normal vector of each leaf surface based on the fitted points using the covariance matrix (Yun et al., 2016). Then, the distance dist^normal^ between the highest point $p_{i,high}^{MLS}$ and the lowest point $p_{i,low}^{MLS}$ in the direction of the calculated normal vector of the leaf blade was selected as the equivalent leaf thickness of the current blade to describe the curl, twist, and droop degrees of the leaf surface. Additionally *dist* ^*normal*^, was set as the height (thickness) and coupled with the hexagonal base mentioned above to constitute a hexagonal prism that can appropriately wrap the fitted point cloud P^LMS^ of a single leaf. The hexagonal base of a hexagonal prism can be divided into three components, namely, an upper triangle, a middle trapezoid, and a lower triangle, along with the equivalent leaf thickness *dist* ^*normal*^. Hence, the equivalent volume of each leaf *V*_*leaf*_*i*__ with varying degrees of leaf distortion based on the wrapped hexagonal prism can be calculated. The experimental diagrams of the hexagonal prisms enclosing the point clouds of different individual leaves are shown in the last row of **Figure 3**, with the black arrows, blue stars and pink stars representing the normal vectors of the leaf blades, the highest points, and lowest points along the direction of the normal vector, respectively.

### Volume-based gap fraction in the tree crown calculation

After the suitable representation of every individual leaf in the tree crown using hexagonal prisms with automatic adaptive parameter assignment, the equivalent volume of every leaf *V*_*leaf*_*t*__ in the tree crown was calculated. Additionally, the wood volume *V*_*branch*_*u*__ was derived based on a set of cylinders in the branch direction with the fitted radii (Huang et al., 2020) mentioned in subsection 2.2. The canopy volume *V*_*canopy*_ was obtained using the 3D alpha shape algorithm (Gardiner et al., 2018) based on the scanned points of vegetative elements above the heights of the lowest branches. According to the definition of the void fraction (porosity) of a porous medium describing the fraction of void space (Hewison and Kuras, 2005), the volume-based GF_vol_ of the tree crown is defined by the following ratio:


(6) $$GF_{vol}=1-\frac{\sum_{t=1}^{N}V_{leaf_{t}}+\sum_{u=1}^{M}V_{branch_{u}}}{V_{canopy}}$$

Formula (6) shows that the volume-based GF_vol_ can be calculated as one minus the ratio of the volume occupied by leaf and wood components to the total canopy volume, where *N* and *M* represent the total numbers of leaves and branch segments in the studied tree crown, respectively.

## Results

### Volume-based gap fraction retrieval for three small trees

**Figure 4:**
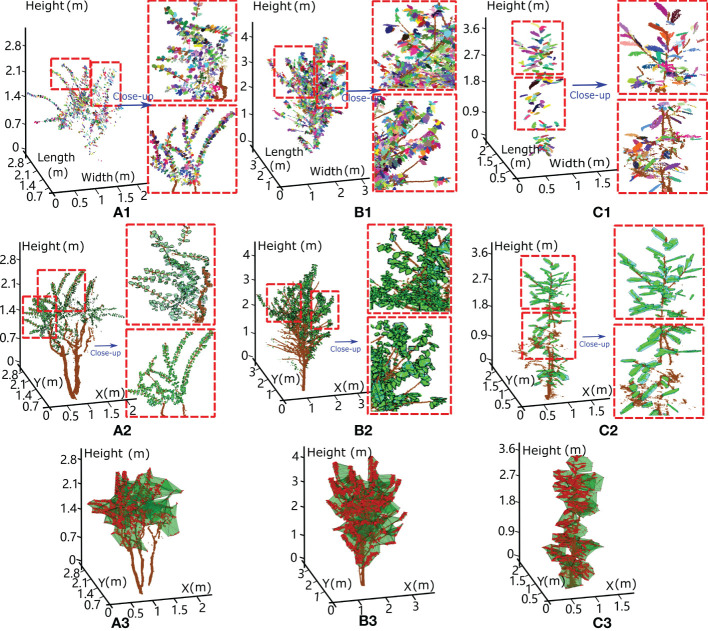
The results for three small experimental trees, with each leaf extracted from the scanned points (a1, b1 and c1) and wrapped with a hexagonal prism (a2, b2 and c2). The tree crown enclosure was determined based on the alpha shape algorithm (a3, b3 and c3) and used to assess the volume-based gap fraction of each tree crown with the proposed method. **(A)** Crepe myrtle tree, **(B)** sakura tree and **(C)** magnolia tree, where the results within the red dotted box are magnified to illustrate the validity of our algorithm.

The stepwise results of our method for three small trees, namely, crepe myrtle, sakura and magnolia, are shown in **Figure 4**, where the first row shows the individual leaf segmentation results for the scanned tree points highlighted in different colours and the second row shows the generation of the hexagonal prism envelope to wrap each individual leaf point. The overall volume of the crown was calculated using the convex hull of the alpha shape algorithm based on the point set of the tree crown, with the results for three tree species shown in the last row of **Figure 4**. **Table 2** lists the detailed results of our approach for the three studied trees.

**Table 2:** The inversion results for the volume-based canopy gap fraction and related branch and leaf parameters for the five experimental tree species.

Tree species	Crepe myrtle	Sakura	Magnolia	Rubber tree	Ginkgo
Number of leaves	1182	2870	354	11633	12654
Leaf length/width (cm)	5.16 ± 2.05/ 3.63 ± 1.32	9.97 ± 3.45/ 5.36 ± 1.83	14.27 ± 5.06/ 6.31 ± 2.47	17.87 ± 6.75/ 7.09 ± 3.15	4.72 ± 1.91/ 7.26 ± 3.28
Equivalent thickness of leaves (cm)	1.02 ± 0.65	1.10 ± 0.75	1.89 ± 1.21	1.68 ± 1.14	1.72 ± 1.48
Equivalent volume of individual leaves (cm^3^)	15.22 ± 6.83	64.81 ± 23.64	144.07 ± 56.81	147.00 ± 78.29	41.13 ± 19.48
Total equivalent volume for foliage/wood (m^3^)	0.018/0.014	0.186/0.063	0.051/0.019	1.71/0.560	0.52/0.101
Crown volume (m^3^)	2.15	5.34	2.18	86.17	14.86
Leaf area index (LAI)	0.81	2.12	1.42	3.70	4.11
Volume-based GF_vol_	0.985	0.953	0.968	0.973	0.958

Crepe myrtle is a small tree with small leaf blades (length of 5.16 ± 2.05 cm and width of 3.63 ± 1.32 cm). Each side of the lamina bends inward from the midrib, leading to a relatively small equivalent leaf thickness (mean of 1.02 ± 0.65 cm). Due to sparsely distributed leaves and spreading of the tree crown, the occupied total leaf equivalent spatial volume (0.018 m^3^) and estimated wood volume (0.014 m^3^) are the smallest among those of the three trees, yielding a small LAI (0.81) and a high volume-based GF_vol_ (0.985). The sakura tree was in the intermediate stage of leaf loss and preparing for dormancy; hence, medium-sized leaves (leaf number of 2870, length of 9.97 ± 3.45 cm and mean width of 5.35 ± 1.83 cm) and a few leafless branches were found in the tree crown. Many curled leaves were droopy, manifesting a medium equivalent leaf thickness of 1.10 ± 0.75 cm and a low volume-based GF_vol_ of 0.953. Magnolia, in an early phase of leaf expansion, has a narrow cylindrical tree crown and larger leaf blades (mean length of 14.27 ± 5.06 cm and mean width of 6.31 ± 2.47 cm). The large magnolia leaf strengthened or supported by fibre bundles is composed of overlapping sclerenchyma cells that form a hard and coarse leaf blade with a relatively twisted lamina, which results in a greater equivalent leaf thickness (mean of 1.89 ± 1.21 cm). Due to the small number of leaves present in the tree crown, the total equivalent leaf volume is 0.051 m^3^, resulting in a moderate volume-based GF_vol_ value of 0.968.

### Volume-based gap fraction retrieval for two tall trees and a forest plot

**Figure 5:**
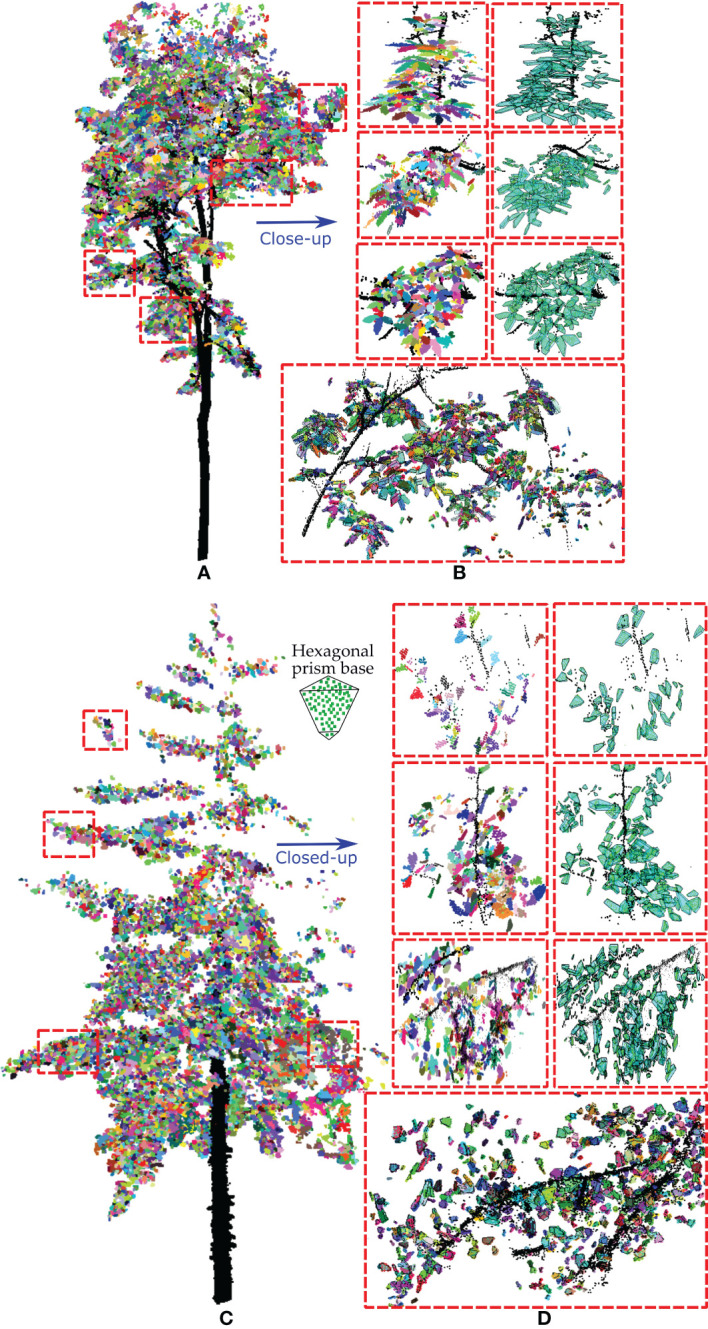
The experimental results of our method applied to the two taller trees. **(A, C)** show the leaf separation results for a rubber tree and a ginkgo tree, respectively, with the segmented individual leaves highlighted in different colours. **(B, D)** Close-up of four local areas in each tree crown displaying the extracted individual leaf points and the corresponding enclosed hexagonal prisms, separately or together.

To test the applicability of our method for tall trees and a dense forest plot, we applied it to one rubber tree of clone CATAS 7-20-59 located within a rubber tree plantation in Danzhou city, Hainan Island, China, and a ginkgo tree on our campus. The detailed growth properties are shown in **Table 1**. Rubber trees are perennial tropical trees and have compound leaves, with each compound leaf usually comprising three leaflets attached to the middle vein but with their own petioles. Compared with the crowns of small trees, those of tall trees contain more leaf blades, yielding more cases of missed data and shading effects when scanned data are collected using terrestrial LiDAR in a bottom-up scanning pattern, which results in decreased accuracy for individual leaf separation and a longer program execution time (approximately 10 minutes). The individual leaf segmentation results for the rubber tree are shown in **Figure 5A**, with the locally magnified results paired with the constructed hexagonal prisms enveloping the points of each segmented leaf shown in **Figure 5B**. Notably, relatively highly accurate segmentation results with geometrically explicit leaflet lamina rendered by hexagonal prisms can be observed. Although subtle over- and undersegmentation issues exist for individual leaflets, the overall visual aspects of the modelling results for the rubber tree leaflets are adequate. **Table 2** shows the result that a total of 11633 leaflets with equivalent leaf thicknesses of 1.70 ± 1.34 cm occupy 1.71 m^3^ of equivalent volume in the large tree crown (volume of 86.17 m^3^), with a wood volume of 0.56 m^3^, from which the volume-based GF_vol_ of the rubber tree crown is derived to be 0.973.

The fan-shaped leaves of the ginkgo are droopy and irregularly notched. They are often deeply grooved in the middle of the leaf, producing two distinct lobes. Ginkgo trees have many fork-shaped juxtaposed fine veins, and this vein arrangement cannot support the leaves well, causing the leaves to fall from the middle to the ends and making the ginkgo leaves appear soft and droopy. The ginkgo tree has a cone-shaped tree crown and clusters of leaves grown near the first- or second-order branches, which results in the occlusion effect dominating in the upper part of the canopy, and terrestrial views are insufficient for the collection of branch data. Therefore, the scanned data quality of the tree crown degenerates with increasing tree height. Due to the unique fan-shaped structure of ginkgo leaves, we used a hexagonal prism base composed of a larger upper triangle, a smaller lower triangle and a middle trapezoid connecting them to align to the leaf blade shape, which is shown in **Figures 5C, D**. As shown in **Figures 5C, D**, a certain degree of reduction in the point density in the upper canopy has some adverse impacts on the leaf segmentation and visualization results using our method. Many leaves identified in different colours with incomplete point clouds caused by occlusion were also appropriately enclosed by hexagonal prisms (bottom image in **Figure 5D**). **Table 2** shows the results obtained by our method, in which a total of 12654 leaves with equivalent leaf thicknesses of 1.72 ± 1.48 cm occupy an equivalent volume of 0.52 m^3^ in the middle-sized tree crown (volume of 14.86 m^3^), with a wood volume of 0.101 m^3^, from which the volume-based GF_vol_ of the ginkgo tree crown is derived to be 0.958.

**Figure 6:**
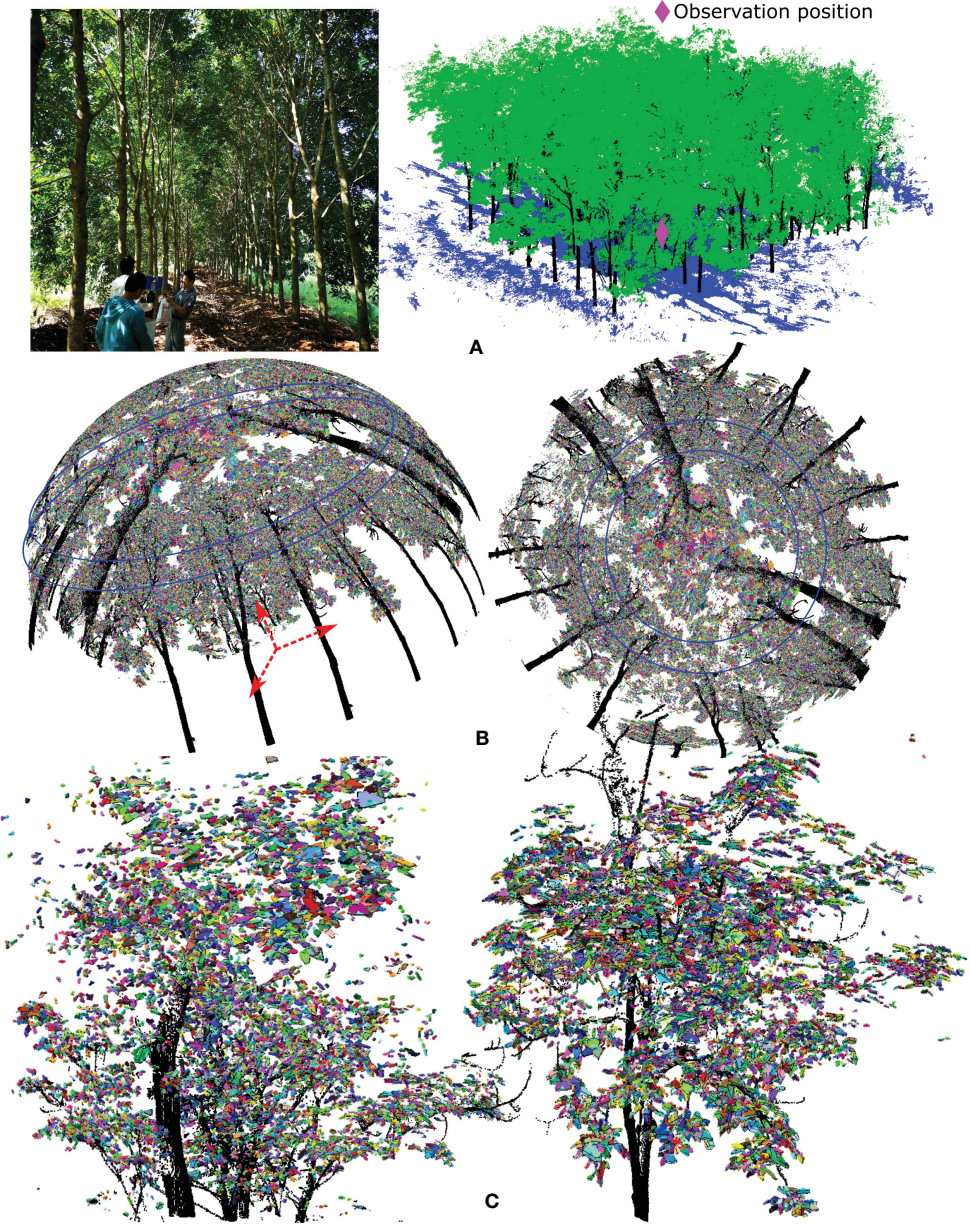
shows the volume-based gap fraction calculation for a rubber tree plot. **(A)** The images and scanned points for a lush forest plot of rubber tree clone PR 107 with interlacing branches and foliage clumps. **(B)** With the ground centre of the plot as the projection centre for the spherical projection, the scanned points after wood–leaf segmentation and individual leaf extraction were transformed to a synthetic hemispherical photo (HP) to assess canopy openness, i.e., to estimate the HP-based gap fraction. **(C)** Magnification of partial tree crowns with identified individual leaves highlighted in different colours and enveloped by translucent hexagonal prisms using our algorithm.

Finally, a forest plot (**Figure 6A**) comprising the 24 rubber trees of clone PR 107 mentioned in Subsection 2.1 was selected to calculate the volume-based GF_vol_. The calculated average tree height and crown volume for each tree were 17.52 ± 3.53 m and 79.47 ± 17.85 m^3^, respectively. The average LAI of the plot was approximately 3.9 at the time of data collection (Chen et al., 2015). After the wood–leaf classification process and individual leaf extraction, the synthetic HP of the forest plot according to the virtual observation position set at the ground centre of the plot was generated using spherical projection of the scanned points, from which the retrieved HP-based GF_img_ was 0.284 at the ring segment labelled by blue circles, with zenith angles ranging from 50 to 60 degrees (**Figure 6B**). The average detected number of leaves, mean calculated total equivalent leaf volume and wood volume for each tree were 12120 ± 2689, 1.75 ± 0.47 m^3^ and 0.63 ± 0.19 m^3^, respectively. Then, the retrieved volume-based GF_vol_ for the whole plot using our approach was 0.970. The point clouds of the whole plot were obtained by registration of many terrestrial scans from different scanning positions, which led to an inhomogeneous point density distribution caused by mutually occluded effects or local areas scanned multiple times. Some detail was lost as a result of the incomplete coverage of anisotropic tree crown shapes by the laser beams as well as the presence of ghost points stemming from foliage jitter caused by the wind. Hence, as seen in **Figures 6B, C**), some leaves in the upper part of the forest canopy with low density and incomplete scanned points yield fragmented geometric approximations, leading to minor overestimation of the volume-based GF_vol_, while certain foliage clumps at lower tree heights with highly clustered leaves and ghost points produce spuriously larger hexagonal prisms, resulting in the subtle underestimation of volume-based GF_vol_. Therefore, we expected minor deviations in the volume-based GF_vol_ for the lush forest plot due to the limitation of the technical specifications of the scanner and the complex forest environment.

### Comparison with existing methods

**Figure 7:**
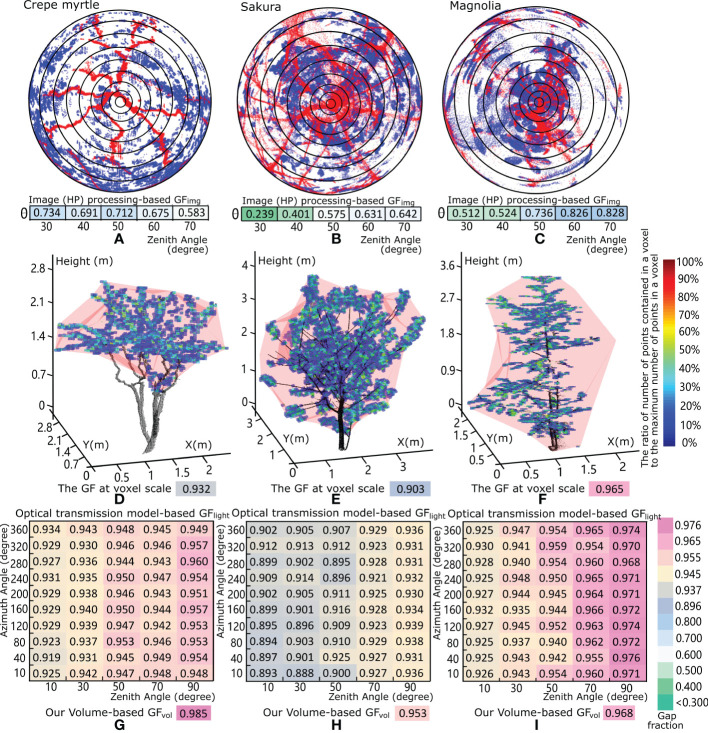
Comparison of the results of our method and two existing methods of gap fraction retrieval for the three studied trees: the crepe myrtle, sakura and magnolia trees. **(A–C)**, Gap fraction GF_img_ calculations from the generated HP through a Lambert azimuthal equal-angle projection of the scanned tree points. **(D–F)** and 3D visualization of a cubic voxel-based representation of the three studied trees, where the different colours of each voxel represent the ratio of the number of scanned points in each voxel to the maximum number of scanned points in the voxel within the tree crown. The gap fraction at the voxel scale, i.e., the canopy volume minus the total volume of all voxels that contain scanned tree points, divided by the canopy volume for each tree crown is shown in **(D, E)**. **(F–I)** show the matrix of gap fraction GF_light_ results for each tree crown based on the optical transmission method, which varies with different azimuth and zenith angles of emitted light. The GF_vol_ values are roughly equal to those of our volume-based method when the zenith angle of the emitted light is large.

Two typical methods of GF retrieval based on HP processing (Zheng et al., 2013) and optical transmission models (Béland et ., 2011) were applied to the three selected experimental trees to perform a quantitative assessment of our results versus those of existing methods. The specific comparison is shown in **Figure 7**. Based on a simulated HP generated from scanned data using the Lambert azimuthal equal-angle projection shown in **Figures 7A–C**, GF_img_, i.e., the ratio of the number of sky pixels to the total number of pixels, was calculated for each annulus sector of the HP corresponding to different zenith angles. The GF_img_ calculated with 20° intervals is shown in **Figures 7A–C**. Usually, GF_img_ at a zenith angle equal to 57.3° is considered the representative index for characterizing the target trees. Here, the GF_img_ magnitudes obtained from the HP at a zenith angle of 57.3° (crepe myrtle 0.693, sakura 0.590 and magnolia 0.762) are smaller than those obtained with our volume-based approach. A reasonable explanation for this difference is that the GF_img_ retrieved from the HP was deduced from the overlap of leaf and wood materials from an upward-looking observation point from the root to the treetop, and this calculation was performed after the conversion *via* the Lambert azimuthal equal-area projection with the original spatial void distribution reduced. Additionally, the intrinsic spatial geometric information of the tree crown was compressed to a two-dimensional domain, with considerable 3D feature loss and limited structural representation. Although qualitatively similar results were obtained with the HP approach, such that the ranking of trees’ GF_img_ remained the same, the methodological differences led to different results.

Another method for GF_light_ estimation (Béland et al., 2011; Béland et al., 2014) is based on voxelization and a model of optical transmission through the canopy. The point clouds of the tree crown were subdivided into numerous cubic voxels. A ray-tracing algorithm that computes the distance of each ray travelling through a tree crown before being intercepted by vegetative elements was used for total leaf area calculation. As shown in **Figures 7D–F**, the GFs at the voxel scale, i.e., the canopy volume minus the total volume of all voxels that contain scanned tree points, divided by the canopy volume indicated by the translucent red convex hull were 0.932 for the crepe myrtle, 0.903 for the sakura tree, and 0.965 for the magnolia tree. Moreover, each voxel was assigned a colour according to the ratio of the number of points contained in the current voxel to the maximum number of points in the voxels of the current tree crown, and most voxels, with blue or light blue colour, represent only 10-30% point inclusion relative to those with the maximum number of points. This finding suggests that the leaf elements are distributed evenly and sparsely in the crown space, and a large proportion of light is not intercepted by the points of foliage elements in each voxel, resulting in a high value for the optical transmission model-based GF_light_. The retrieved GF_light_ magnitudes based on the optical transmission models for the three trees are shown in **Figures 7G–I**, with values roughly equal to those obtained by our volume-based method. The reason for this similarity is that the optical transmission model considers two key factors, namely, the vegetative materials contained in each spatial voxel and the average distance travelled by the emitted laser beams through the canopy as they hit or do not hit vegetative elements, which is related to the tree crown geometry and the properties of foliage elements in each voxel. As heterogeneous tree crowns produce varying degrees of occlusion from different viewing angles, the GF_light_ calculation method based on the optical transmission models yields variable assessment results with changes in the azimuth and zenith angles of the laser-scanning emission positions relative to the target trees. Hence, the results take a two-dimensional matrix form for each tree, as shown in **Figures 7G–I**, with variations in the zenith and azimuth angles. The optical transmission model-based results obtained from the lateral side of each tree, i.e., the vertical profile with a large zenith angle and abundant spatial information, indicate that the sakura tree has the lowest average GF_light_ value (0.934) and that the crepe myrtle has the highest average GF_light_ value (0.953); these findings are qualitatively consistent with our results.

## Discussion

### Different types of methodologies for GF estimation

From the perspective of porous medium theory, GF_vol_ (porosity or void fraction) is the ratio of the volume of voids or pore space to the total volume of the entity (Dutta and Tarafdar, 2003). This variable is widely used in geology, hydrogeology and soil science. The volume-based GF_vol_ is a fraction that varies between 0 and 1, typically ranging from less than 0.005 for solid granite to more than 0.5 for peat and clay (Dutta and Tarafdar, 2003). Other earth substances also have measurable volume-based GF_vol_ values or porosities. For example, for sand, the space among particles exhibits average volume-based GF_vol_ values of 37.7%, 42.3% and 46.3% for packed, natural (in situ) and loose packing conditions (Roman-Sierra et al., 2014), respectively. Expanded polystyrene foam for insulation usually contains approximately 3% polystyrene and 97% stationary air, with a volume-based GF_vol_ of 97% (Yu et al., 2015). Tree crowns grow on Earth with relatively large crown volumes containing thin leaf blades and little woody material. Considering a unified framework for the volume-based GF_vol_ values of various types of porous masses around the world, the porosity of the tree crown is similar to that of expanded polystyrene foam or a sponge, with a high gap fraction magnitude (GF_vol_ >95%). This structure results from a relatively large crown volume containing considerable void space to allow light, air and liquid to pass through; the estimated ranges of the GF_vol_ magnitude for tree crowns and the corresponding effects on these ranges are presented in the Supplementary Materials. Moreover, unlike downhole rock or sediment, sonic and neutron logs are needed to measure the porosity of clay-rich coal rock (McNally, 1990), and unlike industrial and other everyday materials, for which scanning electron microscopy can be applied for porosity quantification (Alvarez et al., 2019), the volume-based GF_vol_ values of tree crowns can be estimated with visual trait heuristic- and computer graphics-driven frameworks. These frameworks, in combination with the dense point cloud data collected by the LiDAR techniques can provide valuable insights into vegetative element modelling and calculations of the spatial structure of tree crowns.

Existing studies of GF retrieval for tree crowns mainly focused on canopy image processing techniques (Gonsamo et al., 2010) or optical transmission algorithms (Kobayashi et al., 2013). Image processing-based methods can be used to derive below-canopy images, e.g., captured digital hemispherical images or synthetic HPs produced from scanned points, and coupled with image segmentation algorithms to calculate the proportion of sky pixels in an image (Hancock et al., 2014). This approach is convenient for GF_img_ calculations at the individual tree or forest plot scale and retains some meaningful properties of the original targets in 3D space. However, with the use of 2D HP images to identify the spatial composition of many natural vegetative materials, considerable detail regarding the intrinsic geometric properties of plant parts in a compressed space is lost.

Optical transmission model-based GF_light_ methods are often built on the foundation of the Bouguer-Beer–Lambert law (Bouguer, 1922) related to the attenuation of light travelling through homogeneous materials. The original Beer law is commonly applied in studies involving chemical analysis or attenuation in physical optics, but the performance of Beer’s law is limited when the medium attenuating light is inhomogeneous and turbid (Regnima et al., 2017), causing multiple scattered photons and requiring correction terms for the Lambert–Beer law (Wind and Szymanski, 2002). Similarly, uncertainties exist when Beer’s law in Formula (7) is used to calculate the GF_light_ *P*(*θ*) of tree crowns as turbid media. Such uncertainties exist for the probability of a beam or a ray of light penetrating the canopy at an incident angle *θ* without being intercepted and other for other factors, as described below.


(7) P(θ)=exp[−G(θ)·Ω·LAI/cosθ]

In Formula (7), the extinction coefficient *G*(*θ*) is based on the range of foliage inclination and azimuthal angles to the incident laser beams, with a magnitude of 0.5 and a standard deviation within 0.1 (Woodgate et al., 2015). The clumping index Ω describes the deviation of the foliage arrangement from a random distribution, of which the magnitude ranges from 0.4 to 1 for coniferous forest (Zhao et al., 2012) and 0.3-0.9 for broad-leaf forest (Woodgate et al., 2015). In general, trees have highly variable phenotypic traits and irregularly discontinuous canopies with anisotropic leaf orientations and multifactor-driven foliage clumping, which hinders the accurate estimation of the magnitude of two key parameters, *G*(*θ*) and Ω, in Formula (7) and indicates the potential for a biased result in the final assessed GF_light_ *P*(*θ*). In addition, the GF_light_ results derived from optical transmission models are affected by the light emission positions and incident angles from a laser scanner with a limited field of view (Li et al., 2017). Any factor fluctuations in the laser beam divergence, scanning resolution and reflectance albedo of varying object surfaces lead to perturbation and error propagation in estimates of the final tree crown GF_light_ (Hancock et al., 2014). Hence, it is meaningful for the volume-based GF_vol_ method based on porous media theory to involve a more comprehensive measurement setup that combines the collected point clouds of the tree canopy and computer graphics algorithms. This approach can be used to identify GF_vol_ as a forest canopy property descriptor for quantitatively assessing the spaces among the vegetation elements in the canopy.

### Merits and limitations of our method

The aim of our approach is to support the shift from 2D image analysis or relatively coarse geometric depiction to fine 3D spatial element rendering for tree crown GF measurements. In recent years, researchers have sought to characterize the phenotypic traits of vegetation and detailed canopy architectures. Some progress has been made, such as the use of regular geometric models, e.g., ellipsoids, umbrellas, cylinders or cones, to simulate tree crowns (Duchemin et al., 2018). Additionally, tree crowns have been treated as pseudo-turbid media to describe their internal contents and to assess solar light radiation (Widlowski et al., 2014), voxel-based representations have been applied to determine the spatial distributions of vegetative tissues (Béland et al., 2014), and images captured from different viewing angles have been used to obtain multiangle profiles for analysing within-crown material clumping (Woodgate et al., 2015). Applied computer graphics techniques have been proposed to support robust algorithms that can achieve individual leaf segmentation from point clouds (Koma et al., 2018; Xu et al., 2019), thus enhancing the ability to reveal the detailed traits of real tree crowns and enabling the separate analysis of individual leaves and branch segments. Additionally, tree crowns have a porous structure, which has a major influence on ventilation and light transmission in the tree canopy. Based on the framework of porous media theory for evaluating natural and human-made substances in the world (De Boer, 1992), it is reasonable to treat the tree crown as a porous medium to calculate GF_vol_. Moreover, the concept of the equivalent thickness of leaf blades was proposed to describe the degrees of leaf curling and drooping and paired with tightly enclosed hexagonal prisms to quantify foliage volumes. The wood volume and crown volume can also be assessed with the fitted cylinder method and convex hull algorithm, respectively. Hence, our method based on porous media theory and computer graphics techniques can be used to inversely calculate the volume-based GF_vol_ of the tree crown, i.e., the ratio of the vacant space in the tree crown to the crown volume. This approach overcomes the restrictions of the existing GF estimation methods, such as image processing-based GF_img_ methods (Hancock et al., 2014), in which the original large forest canopy space (1907.3 m^3^) is compressed into a 2D HP image (**Figure 6B**), which greatly exaggerates the undesirable overlapping effect of plant materials, as well as optical transmission model-based GF_light_ methods (Kobayashi et al., 2013), which are constrained by a limited field of view covering local canopy data from a fixed position and thus provide a narrow perspective on the canopy structure. With further development assisted by deep learning techniques (Lin et al., 2022a), the method described here could be used to depict in detail the actual foliage clumping degree among forest canopies for the quantification of tree architecture heterogeneity. Moreover, this approach could be used to reveal the dynamics of abiotic factors such as air circulation and sunlight transmission through forest canopies comprising varying phenotypic traits for an improved evaluation of plant ecological responses.

Our method has certain shortcomings. The accuracy of the individual leaf separation algorithm will yield certain deviations in the final equivalent leaf volume calculation. A high scanning resolution is needed that guarantees that each leaf covers at least 40 scanned points, i.e., a point cloud sampling spacing of less than 0.5 cm is preferred. If a low scanning resolution or excessive occlusion leads to a very sparse density of the scanned points in the tree crown, it will be difficult for humans to distinguish every leaf by visual inspection, and our method will be unable to effectively segment every leaf. Usually, the density of point clouds is sparser in the intermediate and upper parts of a large tree crown (Han et al., 2022), with the extended beam path inducing an increased likelihood of interception and greater occlusion effects. As a result, some individual leaves are represented by fewer points (< 5 points), yielding considerably degenerated leaf morphologies and, in some cases, not providing sufficient control points to define a hexagonal prism enveloping a leaf. For this case, the algorithm performance decreases with under- or oversegmentation issues, and spuriously large hexagonal prisms enveloping points of many leaves may be created; additionally, isolated incomplete foliage points may not be recognized, leading to detrimental deviations in the final volume-based GF_vol_ calculation results. Fortunately, at present, many stationary terrestrial laser scanners (Wang et al., 2021) of well-known brands, e.g., Leica, RIEGL and FARO, provide mature technologies to meet the density assessment requirements of most analyses. Alternatively, the user can adjust the scanning patterns by decreasing the scanning distance or aligning multiple scans in a parent coordinate system to improve the resolution of the tree point clouds. Moreover, our method is time-consuming relative to image processing-based GF_img_ methods because the processes of individual leaf extraction and hexagonal prism envelope determination require comparatively more time, and a graphics processing unit (GPU) should be used to accelerate the creation and manipulation of the generated fine-scale forest scene models. In addition, for leaves with irregular and complicated shapes, e.g., those of Fatsia japonica and conifer needles, it is not convenient to choose suitable geometric prisms enclosing the leaf blade. Nevertheless, with the advancement of computer hardware (Lin et al., 2022b) with superior graphics processing capabilities and artificial intelligence algorithms that can support forest survey applications, we believe that accurate tree feature recognition and plant phenotyping research (Li et al., 2022) at fine scales will become easier and more accurate in the future, which will propel the development of precision forestry (Holopainen et al., 2014) that incorporates interpretations of the volume-based GF_vol_ of trees.

## Conclusions

This work describes a novel measurement of the volume-based GF_vol_ of tree crowns from TLS data based on computer graphics and porous media theory. The approach is based on the concept of equivalent leaf thickness to describe the universal physiological phenomenon of the degrees of leaf droop and curling. Through individual leaf segmentation and MLS fitting, the equivalent thickness of each leaf surface was calculated from the fitted scanned points associated with the proper-sized hexagonal prism to accurately envelop the points of each leaf and to obtain the equivalent volume of each leaf lamina. Then, the tree crown was considered a multiphase medium, and the total volume of the leaves in the crown and the cylinder fitting-based branch volume were calculated and paired with the assessed crown volume to estimate the volume-based GF_vol_ of each tree crown based on porous media theory. Our calculation method offers a unified evaluation criterion for tree crown GF_vol_ in accordance with the process used to assess the void fractions of most substances in the world. Five trees (three small trees and two taller trees) and a forest plot were selected for experiments, and the calculated volume-based GF_vol_ ranged from 0.95 to 0.98. This magnitude range reflects the many small and thin leaves distributed in a tree crown, promoting less impeded air movement and deep light penetration through the tree crown. Additionally, this study expands the definition of tree crown GF to incorporate the properties recognized by interdisciplinary collaborations and presents an approach to the volume-based GF_vol_ estimation of tree crowns based on the fine phenotypic characteristics inherent in the visual information of plant materials represented by point cloud data. With multidisciplinary advances in the future, the proposed method can be upgraded to provide a deeper understanding of some GF-related plant physiological reactions, e.g., the sunfleck formation mechanism or the preferential paths and resistance of immiscible two-phase flow (light–plant material or air current–plant material) in a porous tree crown, which influence the respiration and photosynthesis of the plant life cycle.

## Data availability statement

The raw data supporting the conclusions of this article will be made available by the authors, without undue reservation.

## Author contributions

YZ: experiment, writing. DL: writing. JF: Programming. XW: Provide data. ME: Control of flow. HZ: proofreading. TY: supervision, writing. All authors contributed to the article and approved the submitted version.

## Supplementary material

Supplementary Figure 1 Several typical crown shapes represented by geometrical primitives, such as **(A)** semi-ellipsoidal, **(B)** ellipsoidal, **(C)** an elliptical cylinder and **(D)** an elliptical cone. Here, it is assumed that the total leaf area of tree crowns can be fully covered by the corresponding geometrical forms with the appropriate sizes and shapes, and this approach was employed to illustrate the derived magnitudes of the LAI and corresponding volume-based gap fraction GF_vol_.
