# Supplementary material for: A reinterpretation of the gap fraction of tree crowns from the perspectives of computer graphics and porous media theory
Source: Front Plant Sci. 2023 Feb 6;14:1109443. doi: 10.3389/fpls.2023.1109443 (PMC9939530; doi:10.3389/fpls.2023.1109443)
Supplement: Supplementary Figure 1 — Several typical crown shapes represented by geometrical primitives, such as (A) semi-ellipsoidal, (B) ellipsoidal, (C) an elliptical cylinder and (D) an elliptical cone. Here, it is assumed that the total leaf area of tree crowns can be fully covered by the corresponding geometrical forms with the appropriate sizes and shapes, and this approach was employed to illustrate the derived magnitudes of the LAI and corresponding volume-based gap fraction GFvol. [file DataSheet_1.docx]

# Supplementary Materials

1 Proof of the rationality of the calculated magnitude range of the volume-based GF_vol_

Our method generates volume-based GF_vol_ magnitudes (approximately 0.97), which are larger than those presented in previous works on canopy gap fractions (Zheng et al., 2013) (Béland et al., 2011). These differences can be accounted for as follows.

In nature, foliage is mainly distributed in the periphery of tree crowns because little light penetrates the intermediate canopy due to blockage by the outermost photosynthetic organs. Moreover, tree crowns usually exhibit several types of shapes (Duchemin et al., 2018), e.g., semi-ellipsoidal, ellipsoidal, elliptical cylindrical and elliptical cones, and the tree crown can be assimilated to the corresponding geometric based on size and shape, as shown in Figure S1. Following assumptions derived from the classical ellipsoidal leaf angle distribution (Campbell, 1986, 1990), i.e., the leaves in a tree crown face all directions with similar probability, these leaves could be moved to the surface of an ellipsoid or 3D geometric primitive representing the tree crown shape and size without large rotation, so as to cover the whole exterior periphery of the geometric primitives, including the upper and lower surfaces. The overall leaf area can be calculated as the surface area of the corresponding 3D geometric primitive. Based on this assumption, the specific tree growth attributes, e.g., total leaf area, crown volume, projection area, leaf area index (LAI) and volume-based GF_vol_, of different crown shapes can be derived from Table S1.


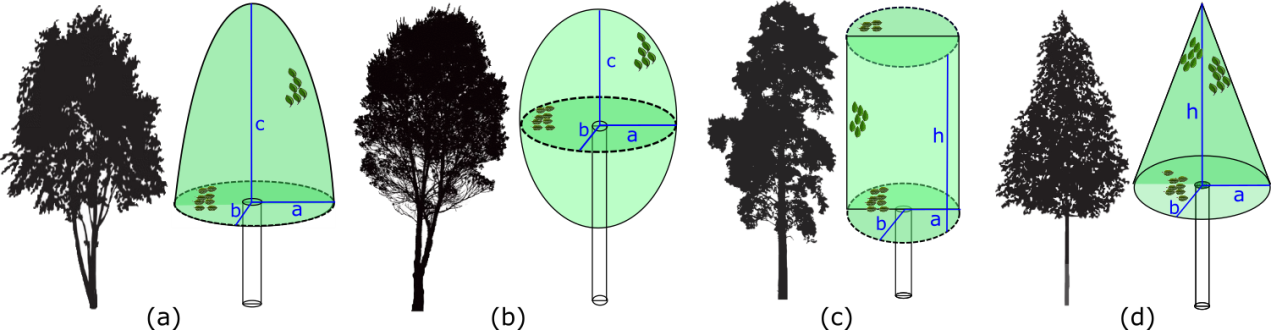


**Fig. S1.** Several typical crown shapes represented by geometrical primitives, such as (a) semi-ellipsoidal, (b) ellipsoidal, (c) an elliptical cylinder and (d) an elliptical cone. Here, it is assumed that the total leaf area of tree crowns can be fully covered by the corresponding geometrical forms with the appropriate sizes and shapes, and this approach was employed to illustrate the derived magnitudes of the LAI and corresponding volume-based gap fraction GF_vol_.

Table S1. Mathematical expression of the growth properties for different tree crowns with shapes similar to various geometric primitives. Here, the true leaf area of the crown is assigned to the surface area of the corresponding 3D geometric primitives, and the average equivalent thickness of the leaf is set to 2 cm.

| Crown shape | Geometry surface area  Total leaf area (m^2^) $surf$ | Crown volume (m^3^) $vol$ | Projection area (m^2^)$projarea$ | LAI | | GF_vol_ | |
| --- | --- | --- | --- | --- | --- | --- | --- |
| Semiellipsoid |  | $\frac{2}{3}\pi abc$ | $\pi ab$ | $\frac{surf}{projarea}$ | $1-\frac{0.02\cdot surf}{vol}$ | |
| Ellipsoid |  | $\frac{4}{3}\pi abc$ |
| Elliptical cylinder | $2\pi ab+\left(2\pi b+4\left(a-b\right)\right)h$ | $\pi abh$ |
| Elliptical cone | $\varpi\text{+}\pi ab$ | $\pi abh/3$ |

Note: $a$ and $b$ represent the semimajor axis and semiminor axis of the ellipse of each geometric primitive, respectively, namely, the half-crown width of each individual tree in two perpendicular directions. $c$ is the radius along the z-axis of the ellipsoid. $c$ and $2c$ represent the crown length (i.e., the tree height minus the clear bole height) for the semi-ellipsoid and ellipsoid tree crowns, respectively. The coefficient $\upsilon\text{=}1.6075$. For the trees with elliptical cylinder or elliptical cone crown shapes, $h$ represents the crown length. $\varpi=\frac{1}{2}\int_{0}^{2\pi} \sqrt{a^{2}b^{2}+h^{2}\left[a^{2}\sin^{2}\left(t\right)+b^{2}\cos^{2}\left(t\right)\right]}dt$.

Here, the average equivalent thickness of the leaf was assumed to be 2 cm, which is larger than the calculated average value for the five target trees (ranging from 1.02 to 1.89 cm) in our manuscript. As shown in Table S2, each tree crown has a total leaf area equal to the corresponding area of the geometric model, resulting in a tightly wrapped tree crown with a large LAI (most of the LAI values are greater than 4). Nevertheless, the corresponding derived GF_vol_ is large, and the tree crown with a flat elliptical cone yields the smallest GF_vol_ of 0.9579 because the cone shape is similar to a [tetrahedron](https://en.wikipedia.org/wiki/Tetrahedron) and has the largest surface-area-to-volume ratio (Harris and Theriot, 2018). Although the woody elements were not incorporated into the GF_vol_ calculation, a tree with a height ranging from 3 to 20 m usually contains 0.03-0.7 m^3^ of lumber according to the standard volume table (Mesavage and Girard, 1956). Hence, relative to a larger crown volume, in this case, the non-photosynthetic tissues only cause a subtle decline in the final GF_vol_ estimation.

In addition, if we use a value of 2 times the total leaf area on the basis of Table S2, then the total leaf area spans the corresponding tree crown twice, with the LAI values of most trees higher than 9, but the yielded magnitudes $GF_{vol}\text{=1-}\left(2\cdot0.02\cdot surf\right)/vol$ are still approximately 0.95, with a minimum GF_vol_ of 0.92 for the flat elliptical cone-shaped tree crowns. The results are in line with the conclusion that the magnitude range of GF_vol_ for tree crowns is approximately equal to the void fraction GF_vol_ of expanded polystyrene foam, which has a similar pore structure that allows the vast storage of air (approximately 97% of the total volume) and contains many small polystyrene beads (approximately 3% of the total volume). This structure is similar to the many small and thin leaves distributed in the large tree crown with abundant void space to allow the passage of solar radiation and ensure air circulation.

Table S2. The calculated leaf area index (LAI) and volume-based gap fraction GF_vol_ for different types of tree crowns under the assumption that (1) the equivalent thickness of the leaf is equal to 2 cm and (2) the total leaf area of all foliage in the crown is equal to the surface area of the corresponding geometric primitive that has the same size and shape as the target crown

| Average equivalent thickness of leaves: 2 (cm) | | | | | | | | | |
| --- | --- | --- | --- | --- | --- | --- | --- | --- | --- |
| Crown shape | Tree crown type | $a$ (m) | $b$ (m) | $c$ (m) | $h$ (m) | Total leaf area (m^2^) | Crown volume (m^3^) | LAI | GF_vol_ |
| Semi-ellipsoid | Small | 4 | 3 | 5 |  | 137.45 | 125.66 | 3.6 | 0.9781 |
|  | Large | 6 | 5 | 12 |  | 444.77 | 753.98 | 4.72 | 0.9882 |
|  | Flat | 6 | 7 | 3 |  | 309.34 | 263.89 | 2.34 | 0.9766 |
|  | Slender | 3 | 2 | 7 |  | 109.97 | 87.96 | 5.83 | 0.9750 |
| Ellipsoid | Small | 4 | 3 | 3 |  | 138.93 | 150.80 | 3.69 | 0.9816 |
|  | Large | 6 | 5 | 7 |  | 450.78 | 879.65 | 4.78 | 0.9898 |
|  | Flat | 6 | 7 | 2 |  | 314.39 | 351.86 | 2.38 | 0.9821 |
|  | Slender | 3 | 2 | 5 |  | 134.81 | 125.66 | 7.15 | 0.9785 |
| Elliptical cylinder | Small | 4 | 3 |  | 5 | 189.65 | 188.50 | 5.03 | 0.9799 |
|  | Large | 6 | 5 |  | 12 | 613.49 | 1131.02 | 6.51 | 0.9892 |
|  | Flat | 6 | 7 |  | 3 | 383.84 | 395.84 | 2.91 | 0.9806 |
|  | Slender | 3 | 2 |  | 7 | 153.66 | 131.95 | 8.15 | 0.9767 |
| Elliptical cone | Small | 4 | 3 |  | 5 | 104.51 | 62.83 | 2.77 | 0.9667 |
|  | Large | 6 | 5 |  | 12 | 322.14 | 376.99 | 3.41 | 0.9829 |
|  | Flat | 6 | 7 |  | 3 | 277.48 | 131.95 | 2.10 | 0.9579 |
|  | Slender | 3 | 2 |  | 7 | 77.08 | 43.98 | 4.09 | 0.9649 |
